# Increased Numbers of Circulating CD8 Effector Memory T Cells before Transplantation Enhance the Risk of Acute Rejection in Lung Transplant Recipients

**DOI:** 10.1371/journal.pone.0080601

**Published:** 2013-11-13

**Authors:** David San Segundo, María Ángeles Ballesteros, Sara Naranjo, Felipe Zurbano, Eduardo Miñambres, Marcos López-Hoyos

**Affiliations:** 1 Immunology Service. Marqués de Valdecilla Hospital, Santander, Spain; 2 Intensive Care Unit, Marqués de Valdecilla Hospital, Santander, Spain; 3 Thoracic Surgery Unit, Marqués de Valdecilla Hospital, Santander, Spain; 4 Pneumology Service. Marqués de Valdecilla Hospital, Santander, Spain; New York University, United States of America

## Abstract

The effector and regulatory T cell subpopulations involved in the development of acute rejection episodes in lung transplantation remain to be elucidated. Twenty-seven lung transplant candidates were prospectively monitored before transplantation and within the first year post-transplantation. Regulatory, Th17, memory and naïve T cells were measured in peripheral blood of lung transplant recipients by flow cytometry. No association of acute rejection with number of peripheral regulatory T cells and Th17 cells was found. However, effector memory subsets in acute rejection patients were increased during the first two months post-transplant. Interestingly, patients waiting for lung transplant with levels of CD8^+^ effector memory T cells over 185 cells/mm^3^ had a significant increased risk of rejection [OR: 5.62 (95% CI: 1.08-29.37), p=0.04]. In multivariate analysis adjusted for age and gender the odds ratio for rejection was: OR: 5.89 (95% CI: 1.08-32.24), p=0.04. These data suggest a correlation between acute rejection and effector memory T cells in lung transplant recipients. The measurement of peripheral blood CD8^+^ effector memory T cells prior to lung transplant may define patients at high risk of acute lung rejection.

## Introduction

The potential success of lung transplantation is limited by the relative high incidence of acute rejection (AR) within the first year of transplantation[1]. Those transplant recipients suffering acute rejection have poor 1 year survival and an AR episode increases the incidence of chronic rejection in the form of bronchiolitis obliterans syndrome [2,3], and BOS is the major cause of mortality after lung transplantation[1,4]. However, the underlying mechanisms for chronic graft deterioration are not clearly understood[5]. 

The alloresponse against the graft could be driven by several effector subpopulations. Thus, knowledge of effector and regulatory mechanisms in alloresponse may help to monitor solid organ transplant recipients.

In addition, lung transplant recipients (LTR), are at high risk of infection, and the immune response against microorganisms can overlap with the alloresponse. The challenge is to differentiate between the donor specific alloresponse and the response against respiratory pathogens. In several transplant settings, regulatory T cells (Tregs) have been demonstrated to play a role in controlling alloresponses in animal models[6], although the transfer to human solid organ Tx gives contradictory results. In liver Tx high Treg levels are associated with tolerance[7] but in other solid organ Tx such an association is less clear[8]. Importantly effector memory subpopulations are able to break the tolerance induced by Tregs[9] and memory alloresponse can be involved in chronic rejection[10] and aggressive AR [11]. In early 90s in vitro studies showed indirect evidence that switch from naïve to primed/memory CD8^+^ T cells was important in kidney allograft rejection[12]. In moderate AR in cardiac allograft biopsies high levels of infiltrating memory subsets has also been shown [13]. In lung-Tx models a role for CD8^+^ T cells in chronic rejection has also been demonstrated [14].

The present study addressed the kinetics in peripheral blood of the number of different effector and regulatory subpopulations in LTR within the first year of transplantation.

## Materials and Methods

### Patients and blood sampling

 A prospective single center study was designed and approved by local Ethic Committee (Ethic Committee of Clinical Research of Cantabria).

Twenty-seven consecutive LTRs followed at our Hospital during 2010 were recruited for the study and 16 sex- and age-matched healthy subjects were gathered as control group. All patients gave their written informed consent. 

The demographic, clinical and main immunological variables are summarized in Table 1 and comparison with control group in Table 2.

**Table 1:** Demographic, clinical and immunological variables of patients included in the study.

	**N**	**Mean ± SD**	**%**
Donor age (years)	14	44±19.2	
Recipient age(years)	27	56.4±10.8	
Sex (M/F, % of F)	17/10		37
Disease			
- Pulmonary Fibrosis	7		
-COPD	11		
-alpha1-antitrypsin deficiency	3		
-Cystic Fibrosis	2		
-Pulmonary sarcoidosis	1		
-Histiocytosis-X	1		
A-Mismatches	13	1.6	
B-Mismatches	13	1.8	
DR-Mismatches	13	1.4	
Post-Tx treatment (tacrolimus+Steroids+MMF)	27		100
Maintenace treatment (tacrolimus+Steroids+MMF)	27		100
Biopsy proven AR	13		48.1
Infections	16		59.3
De novo diabetes	2		7.4

**Table 2:** Comparison of percentage of memory CD8+ T cells of lung transplant recipients with sex- and age-matched healthy controls.

	**Healthy controls**	**Lung transplant recipients**	**P value**
N	16	27	
Age (median and interquartile range)	55 (47-61)	59 (55-62)	NS^a^
Sex (M/F) (% of female)	10/6 (38)	17/10 (37)	NS^b^
% of CD8+ TCM (median and interquartile range)	10.7 (6.95-17.40)	10.1 (6.70-15.40)	NS^a^
% of CD8+ TEM (median and interquartile range)	31.4 (17.39-42.25)	25.6 (14.1-39.2)	NS^a^

U Mann-Whitney^a^ and Chi-square^b^ statistical tests were applied

The patients were monitored and peripheral blood samples were obtained just before Tx, and after 7, 14, 30, 60, 90, 180 and 360 days post-Tx. All recipients were treated with the same immunosuppression regimen: tacrolimus, steroids and mycophenolate mofetil. Transbronchial biopsy protocol at day 21 post-Tx was performed in each patient and AR episode was defined by histopathological diagnosis according to The ISHLT Lung Study Group criteria[15]. Within the AR group, one patient suffered two AR events (1 and 3 months post-Tx) and median time to AR was 30 days post-Tx. The comparison of immunological and clinical data of LTRs included in the groups of AR and AR-free are shown in Table 3. 

**Table 3:** Comparison of demographic, clinical and immunological variables in lung transplant recipients suffering acute rejection episodes and rejection-free.

	**Rejection-free**	**Acute Rejection**	**P value**
Donor age (years; median and interquartile range)	45 (23-61)	46,5 (27-68.3)	NS^a^
Recipient age (years; median and interquartile range)	59 (55-62)	60 (54.5-62)	NS^a^
HLA-A mismatches (mean ± standard deviation)	1.67±0.516	1.57±0.535	NS^b^
HLA-B mismatches (mean ± standard deviation)	2.0±0	1.71±0.488	NS^b^
HLA-DR mismatches (mean ± standard deviation)	1.2±0.837	1.67±0.52	NS^b^
Peritransplant infection (patients with infection/total patients)	1/15	2/13	NS^c^
Infection whithin the first year post-lung transplantation (patients with infection/total patients)	8/15	8/13	NS^c^

*U* Mann-Whitney^a^, *t* Student^b^ and Chi-square^c^ statistical tests were applied

### Flow cytometry studies

At each time point mentioned above, flow cytometry was used to quantify peripheral blood effector and regulatory subpopulations, as described previously [16]. Briefly, whole blood staining with monoclonal antibodies, red blood cell lysis and further wash with Phosphate Buffer Saline for surface staining and intracellular Foxp3 staining (eBioscience, San Diego, CA) were performed following manufacturer’s instructions. The list of antibodies used was: CD62L-fluorescein isothiocyanate (FITC) clone Dreg56, CD45RO-phycoerythrin (PE) clone UCHL1, CD8-peridinin chlorophyll protein (PerCP)-Cy5.5 clone SK1, CD4-allophycocyanin (APC)-Cy7 clone SK3, CD3-PE-Cy7 clone SK7, CD25-APC clone 2A3, CD27-FITC clone M-T271, CD25-PE clon 2A3 (BD Biosciences, San Jose, CA) and CD127-PE-Cy7 clone eBioRDR5 and Foxp3-APC clone PCH101 (eBioscience). Regulatory T cells were defined as CD4^+^CD25^+^CD127^-/low^Foxp3^+^CD27^+^, whereas four different T cell subpopulations based on CD62L and CD45RO staining were defined [17]: naïve (CD45RO^-^CD62L^+^), effector memory (CD45RO^+^CD62L^-^), central memory (CD45RO^+^CD62L^+^) and terminally differentiated effector memory (CD45RO^-^CD62L^-^) T cells in both CD8^+^ and CD4^+^ T cells. All the samples were acquired on a FACSCanto II (BD Biosciences) and analyzed with FACSDiva software (BD Biosciences).

### Blood culture for IL-17 detection

Whole blood cultures were performed for intracellular and supernatant interleukin (IL)-17 measurement.

For intracellular detection, whole blood sample was stimulated with phorbol myristate acetate (25ng/mL) and Ionomycin (1ug/mL) (Sigma-Aldrich, St. Louis, MO), and incubated during 4 hours at 37°C in 5% CO_2_ atmosphere. To avoid cytokine release intracellular transport was stopped by co-incubating with Brefeldin-A (10ug/mL). Surface staining with CD8-FITC/CD69-PE/CD3-PerCP combined antibodies (BD Bioscience), subsequent fixation and permeabilization (BD Bioscience) steps prior to intracellular cytokine staining for IL-17 was performed. 

At the same time, 1:5 diluted whole blood was cultured with 1mg/mL of Concanavalin A (Sigma-Aldrich) for 48 hours at 37°C in 5% CO_2_ atmosphere and supernatant was collected and stored at -80°C until further analysis. The IL-17 levels on supernatants were measured by ELISA following the manufacturer’s instructions (R & D Systems, Minneapolis, MN).

### Statistical analysis

Data were non-parametrically distributed (Kolmogorov–Smirnov fit test) and expressed as the median and the interquartile range. Differences in the percentage and absolute number of Treg, naïve and memory T cells and the expression of different markers between different time points of follow-up were analyzed by Kruskall-Wallis. To compare medians between AR and AR-free groups the Mann-Whitney U test was used. To define a cut off value of CD8^+^ TEM cells to discriminate AR, a receiver operative characteristics (ROC) curve was performed. Univariate logistic regression analysis was used to select factors associated with AR for the inclusion in subsequent multivariate analysis (Table 4). Confounding and collinearity between the selected variables were assessed and finally the model was corrected for age and gender. The p-values <0.05 were considered significant. The data were analyzed using SPSS version 15.0 (SPSS Inc; Chicago, IL, USA).

**Table 4:** Odds ratio for Acute Rejection using a logistic regression.

**Parameter**	**Univariate Analysis**	**P**	**Multivariate Analysis^a^**	**P**
	**OR (95% CI)**		**OR (95% CI)**	
**Recipient Age at Tx**	1.56 (0.34 to 7.11)	0.568		
**Gender (male vs female)**	0.47 (0.10 to 2.29)	0.345		
**Pulmonary disease (fibrosis vs others)**	3.14 (0.59 to 16.84)	0.173		
**Infection**	2.36 (0.19 to 29.71)	0.496		
**CD8+ TEM**	5.62 (1.08 to 29.37)	0.041	5.89 (1.08 to 32.24)	0.041

Tx: TransplantationTEM: Effector memory T cells

a Adjusted for age/gender

## Results

### Prospective follow-up in lung transplant recipients: Tregs

**Figure 1:**
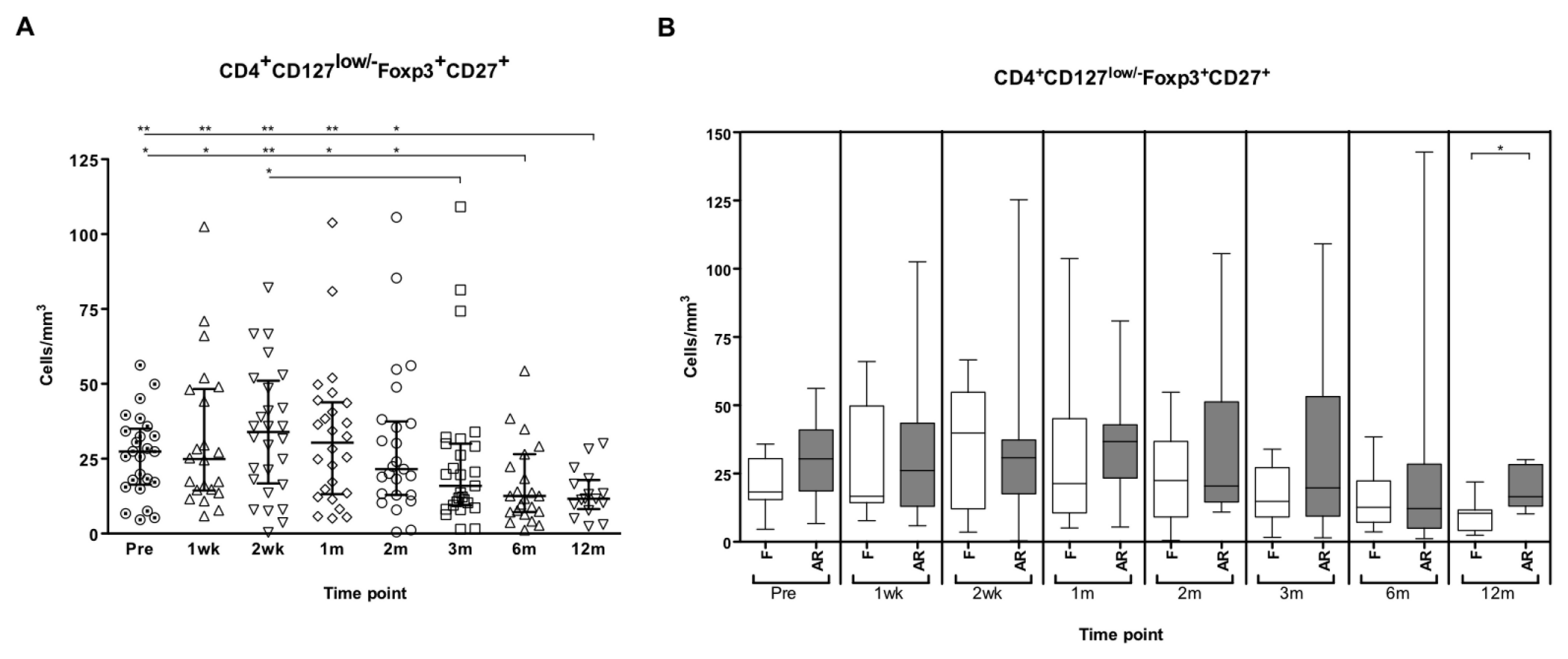
Follow-up of regulatory T cells in lung transplant recipients (LTR). Absolute number of regulatory T cells (CD4^+^CD127^low/-^Foxp3^+^CD27^+^) was measured before the transplant (Pre) and first, second week (wk), first, second, third, sixth, twelfth month (m) post-transplant in peripheral blood of lung transplant recipients (A), median and interquartile range are depicted. (Comparison of absolute number of Tregs in LTR (B)), the box plot shows the median and interquartile ranges of regulatory T (Treg) cells in peripheral blood of lung transplant recipients with acute rejection (AR) episode (grey boxes) and lung transplant recipients without rejection (F, white boxes). The whiskers show 5 and 95 percentile. Kruskall-Wallis and *U*-Mann-Whitney test were assessed to compare medians of Treg levels at different timepoints in AR and F groups, (*, p<0.05 and **, p<0.01).

The absolute number of Tregs in LTRs increased early post-Tx by second month, but significantly decreased in subsequent time points after 6 and 12 months post-Tx (Figure 1A). The patients suffering from an AR event showed increased number of Tregs in all timepoints, but they were only significantly increased at 12 months post-Tx (Figure 1B).

During infections LTRs had similar levels of Tregs compared with infection-free LTRs (data not shown). 

### Th17 cells follow-up in lung transplant recipients

**Figure 2:**
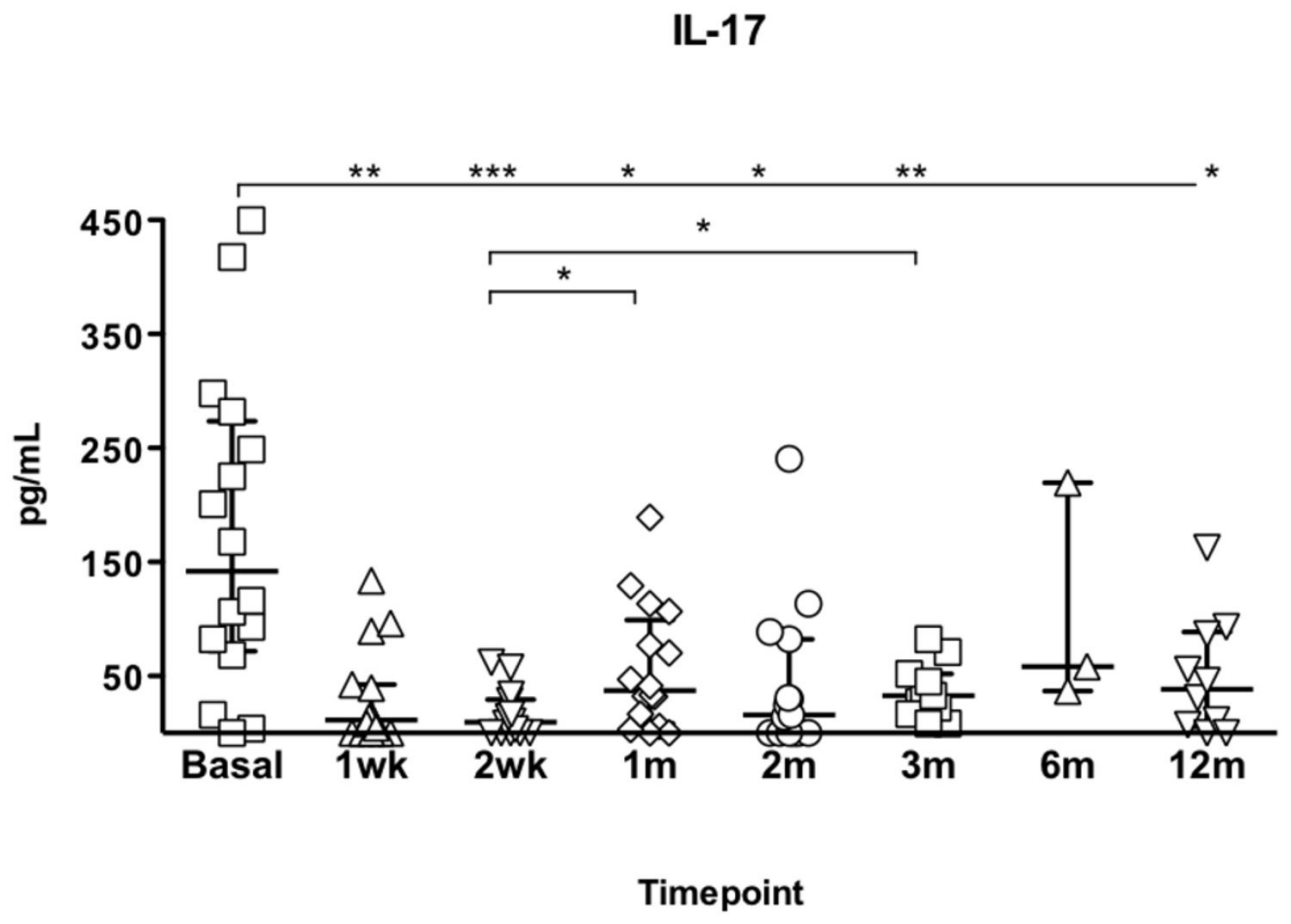
IL-7 measurement in supernatant after 48hour-culture in Lung transplant recipients. Medians and interquartile ranges are depicted and Kruskall-Wallis test was used to compare medians.***p<0.001,** p<0.01, *p<0.05.

No differences in circulating Th17 cell number were observed between AR and AR-free patients.A significant decrease of IL-17 in vitro production was observed at all times point post transplantation compared to pre-Tx levels (Figure 2). However, no difference in IL-17 supernatant levels was observed between the patients with AR and AR-free.

### Memory T cell subsets in lung transplant recipients

**Figure 3:**
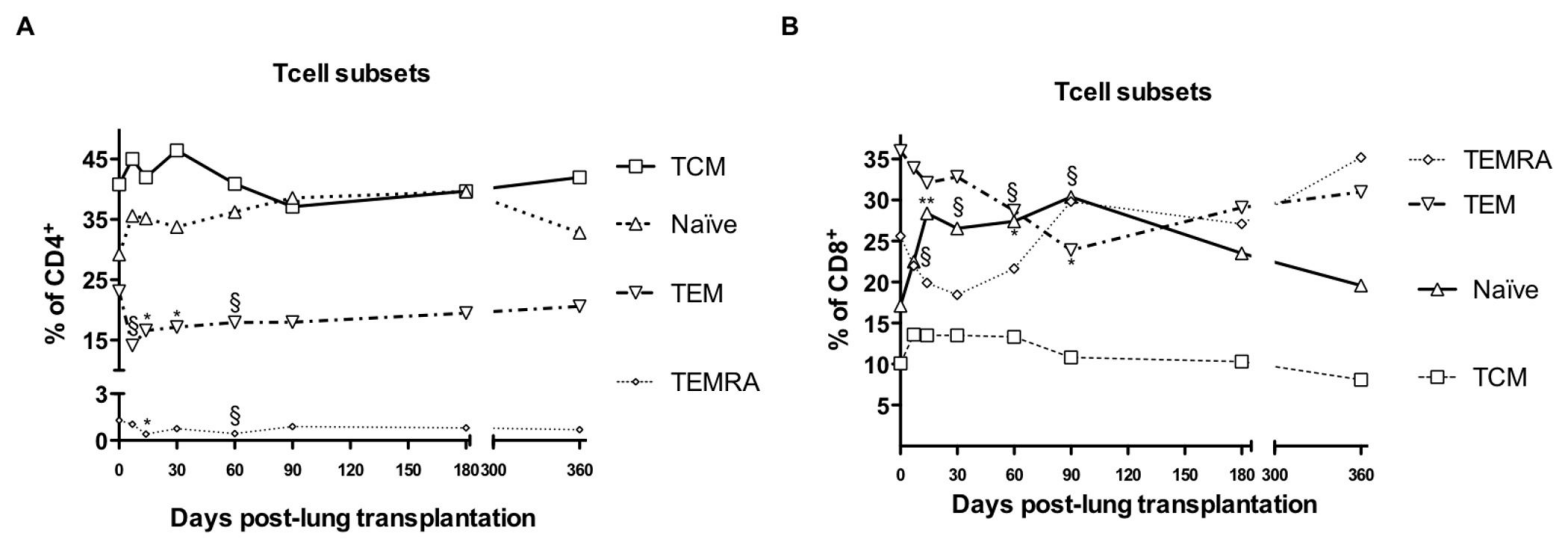
Follow-up of the percentage of T cell subsets. Follow-up of the percentage of CD4+ subsets in lung transplant recipients within first year (A). The median of central memory (TCM) on black line and open squares, naïve on dotted line and open triangle, effector memory (TEM) on truncated line and open triangle and terminally differentiated effector memory (TEMRA) cells on thin dotted line and open diamond are depicted. Follow-up of the percentage of CD8+ subsets in lung transplant recipients within first year (B). The median of central memory (TCM) on black line and open squares, naïve on black line and open triangle, effector memory (TEM) on truncated line and open triangle and terminally differentiated effector memory (TEMRA) cells on thin dotted line and open diamond are depicted. Ranges are not depicted because of simplicity. Median percentage of T cell subset differences were tested by U-Mann Whitney test (* and §, p<0.05 and p<0.1 respectively).

A significant decrease in the percentage of CD4^+^ effector memory (TEM) and terminally differentiated effector memory (TEMRA) T cells, early post-Tx was observed. Such a decrease was maintained during the first month post-Tx and was correlated with an increase in the percentage of CD4^+^ central memory T cells (TCM) (Figure 3A).

The percentage of CD8^+^ naïve T cells increased during the first 3 months of transplantation and recovered to basal levels at 12 months post-Tx. However, CD8^+^ TEM decreased reaching a nadir at 3 months post-Tx with partial recovery at 12 months (Figure 3B). The pattern described above for CD4^+^ TCM was also observed on CD8^+^ TCM. The CD8^+^ TEMRA subset decreased slightly after Tx with gradual recovery from the second month post-Tx.

During the follow-up of the study, a significant increase in both CD4^+^ and CD8^+^ naïve T cells at 2 month post-transplant was observed compared to pre-Tx levels. The CD8^+^ TEMRA cells prior lung Tx were significantly increased compared with the first and second week post-Tx. No differences in absolute numbers of other memory peripheral blood subpopulations at any time point were observed (data not shown).

### Memory T cell subsets and lung graft acute rejection

**Figure 4:**
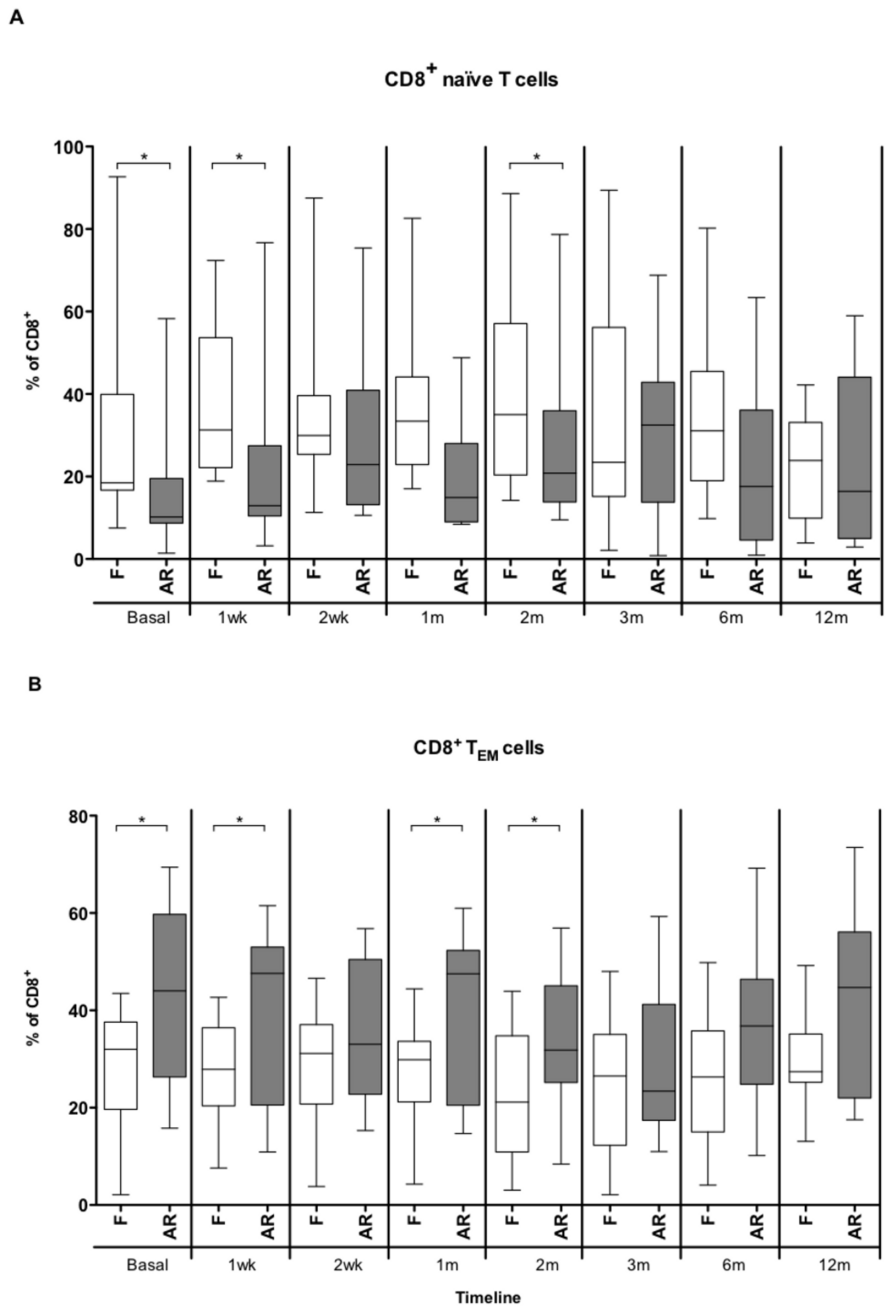
Percenteage of naïve and effector memory CD8+ T cells in lung transplant recipients. Comparison of the percentages of naïve (A) and effector memory (TEM) CD8+ T cells (B) between the groups of rejection-free (F, white box-plot) lung transplant recipients and with an episode of acute rejection (AR, grey box-plot) during several time points post-Tx: pre-Tx (basal), 1 week (wk), 2 weeks, and 1, 2, 3, 6, and 12 months (m) post-Tx. The medians and interquartile range are depicted and compared using Mann-Whitney U test.* p value <0.05.

**Figure 5:**
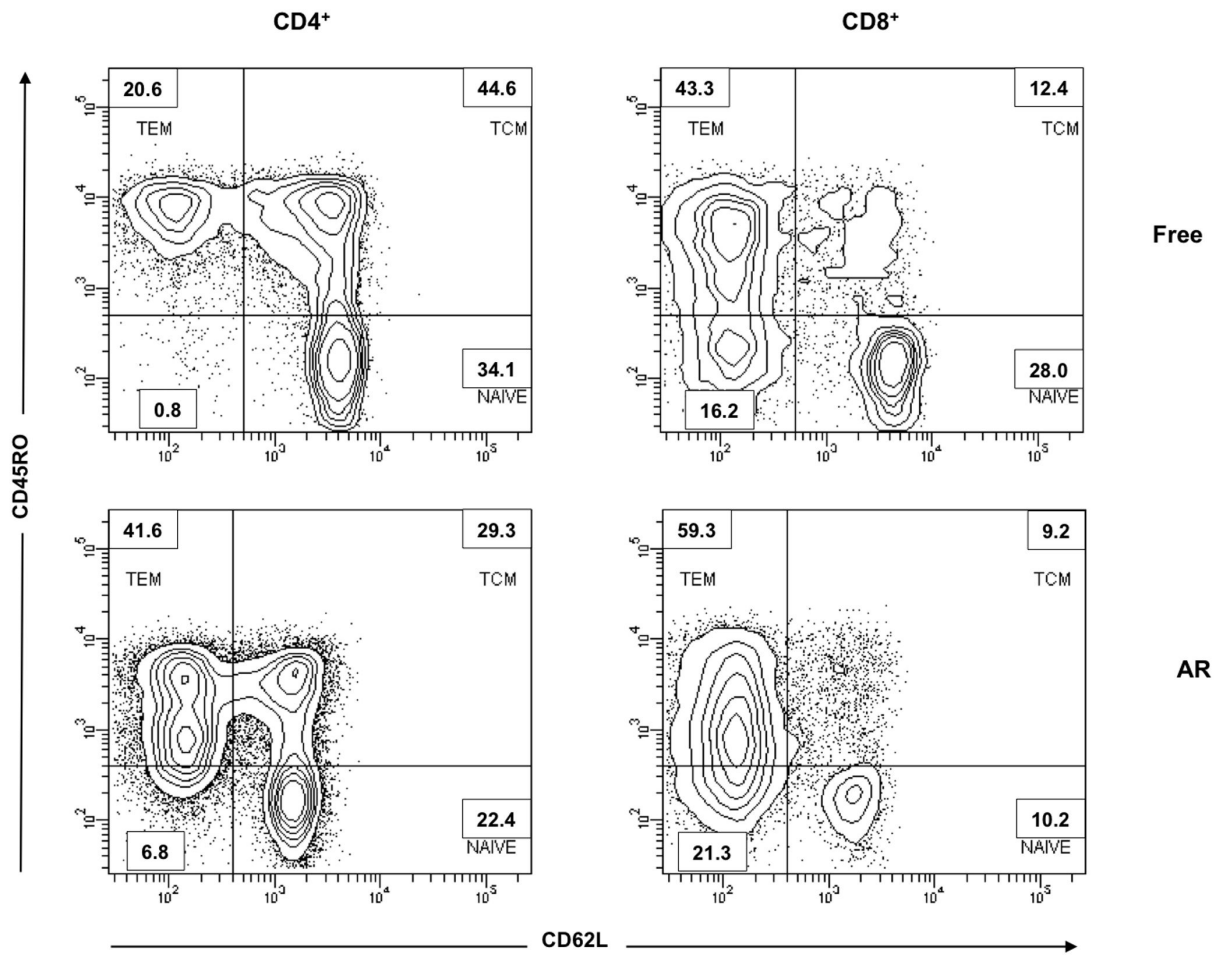
Density-plots of memory T cell subsets in lung transplant recipients. Representative density-plots of acute rejection-free lung transplant recipients (A) and suffering acute rejection episode (B) showing the different subpopulations of CD4^+^ and CD8^+^ T cells before transplantation. Four different subpopulations are depicted: naïve (CD62L+CD45RO-), TCM (CD62L+CD45RO+), TEM (CD62L-CD45RO+) and TEMRA (CD62L-CD45RO-).

In the CD4^+^ subsets no differences in the percentage of TCM, TEM, TEMRA and naive subsets were observed in LTR suffering AR as compared with the AR-free group. A significant increase of CD8^+^ TEM was observed during all the follow-up in the AR group and there was a simultaneous fall in the CD8^+^ naïve subpopulation (Figure 4 and Figure 5).

**Figure 6:**
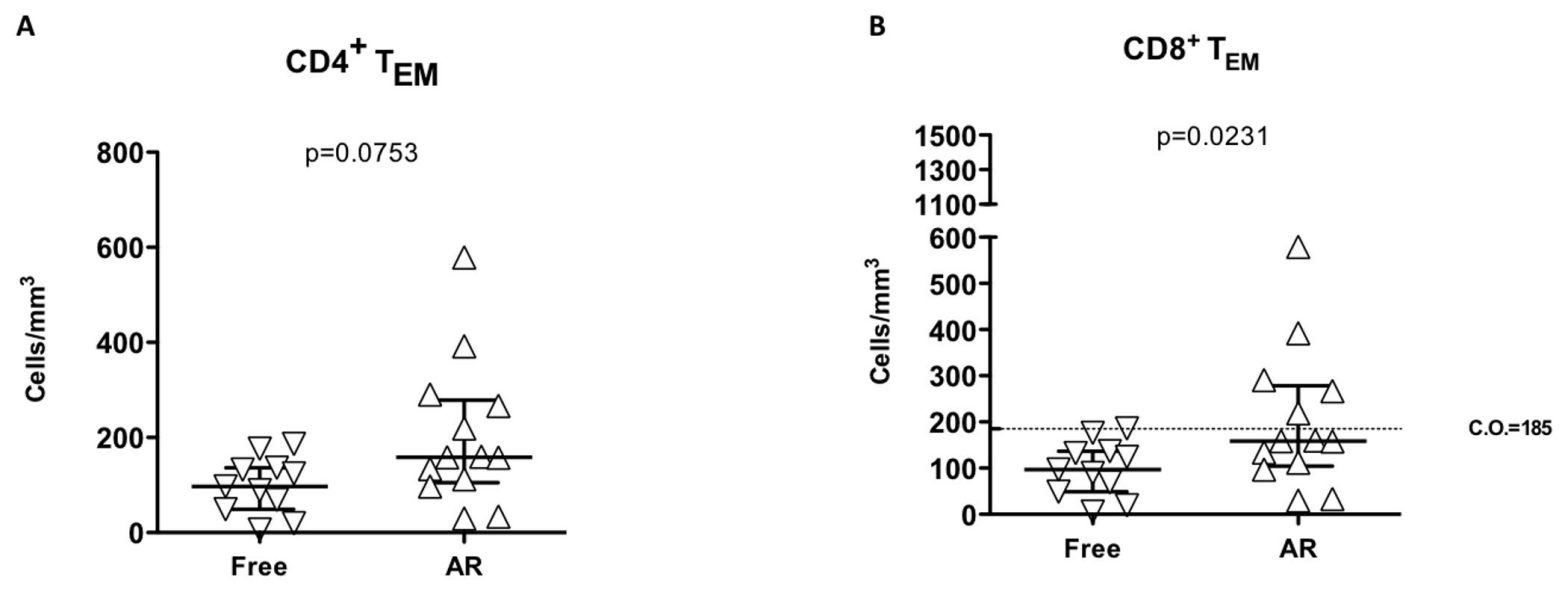
Comparison of absolute numbers of effector memory (TEM) T cells in LTRs. The CD4^+^ (A) and CD8^+^ (B) TEM cells were measured in peripheral blood of rejection-free (Free) and lung transplant patients suffering an acute rejection episode (AR). Medians and interquartile ranges are depicted and compared using Mann-Whitney U test. The cut-off (C.O.) value of 185 CD8^+^ TEM cells/mm^3^ discriminate between Free and AR lung transplant recipients.

In terms of absolute numbers an increase of CD4^+^ and CD8^+^ TEM before transplantation in the AR group was observed (Figure 6).

Using ROC curves to calculate a cut-off value for the number of CD8^+^ TEM to discriminate between AR and rejection-free recipients, 185 CD8^+^ TEM cells/mm^3^ was established as cut-off value. Such a cut-off reached a sensitivity of 69.2% and specificity of 90.9% for predicting a subsequent rejection episode.

The relative risk for AR in patients on the waiting list for lung Tx with CD8^+^ TEM higher than 185 cells/mm^3^ before transplantation was 5.62 CI (1.08-29.37) (p=0.041). We assessed clinical and immunological variables potentially involved in acute rejection in a logistic regression model and corrected them for age and gender (Table 4). The CD8^+^ TEM cells before lung Tx achieved an odds ratio of 5.89 CI (1.08-32.24) (p=0.041).

## Discussion

The effector mechanisms involved in allo-responses are complex and only partially understood. Tregs have gained importance in transplantation due to the findings of their ability to efficiently control alloimmune responses. The findings in LTR however are contradictory. Several studies have correlated low Treg levels in bronchoalveolar lavage [8] with development of AR and BOS[18,19]. Another study found no correlation between frequency of Tregs and BOS outcome, although a role of CCR7^+^CD45RA^-^ Tregs in protection against development of BOS was observed[20].

In the current study lung transplant recipients showed little change in Tregs in peripheral blood over the first year of transplantation and there was no change in those with AR . Our results confirm the lack of association of peripheral blood Treg levels with AR and lung pathology shown by others[21,22]. Although an association between immunosuppressant regimen and Tregs has been demonstrated in other solid organ transplants[23,24], in our cohort no correlation with TAC levels and Tregs was observed at any time point (data not shown).

Despite TCM cells seem to be more resistant to depletion after induction therapy with Campath-1H [25], the impact of several immunosuppressants in memory T cells remain to be fully elucidated. In a different retrospective study of living donor renal recipients after alemtuzumab induction AR inferred an increased proportion of CD4^+^ TEM and CD8^+^ TEMRA 3 years post-Tx[26]. No prospective data on lung transplant patients and memory subsets have been performed. In the present study all the LTR were under the same immunosuppressive regimen without induction therapy, thus the potential impact of induction immunosuppression on memory T cells is avoided.

Within effector subsets, Th17 cells may be involved in allograft rejection in animal models[27] and IL-17 has been associated to the development of BOS in LTR[28]. In our cohort no correlation of Th17 cells, measured by either intracellular or supernatant secretion of IL-17, with AR was observed. Furthermore, different ratios of effector subsets (TEM, TCM, Th17) versus Tregs or naïve T cells in blood were assessed but none of them achieved statistical significance (data not shown).

Our data point to an increased number of CD8^+^ TEM before Tx in patients who later developed an AR episode. The differences were still significant after 2 months post-Tx. This observation was not accompanied with increased production of interferon-gamma or IL-17 after polyclonal stimulation in LTR with AR. More importantly, the patients with end-stage lung disease with CD8^+^ TEM cells higher than 185 cells/mm^3^ presented a substantial increased risk of suffering AR episode. The present study is the first showing a direct association of high levels of pre-Tx TEM cells and AR risk in LTR (Figure 6). There are few attempts in solid organ transplantation to point out memory T subsets as inducers of AR[29,30]. The main limitation of the study is the sample size and the results should be interpreted carefully, and larger multicenter studies should be designed to confirm our data. From our results, the measurement of peripheral blood CD8^+^ TEM cells could be of interest to detect patients before Tx with a potential increased risk of suffering an episode of AR and potentially alter induction regimens for such patients. 
